# A Novel Wearable Device for Motor Recovery of Hand Function in Chronic Stroke Survivors

**DOI:** 10.1177/1545968320926162

**Published:** 2020-05-26

**Authors:** Supriyo Choudhury, Ravi Singh, A. Shobhana, Dwaipayan Sen, Sidharth Shankar Anand, Shantanu Shubham, Suparna Gangopadhyay, Mark R. Baker, Hrishikesh Kumar, Stuart N. Baker

**Affiliations:** 1 Institute of Neurosciences, Kolkata, West Bengal, India; 2 Newcastle University, Newcastle upon Tyne, Tyne and Wear, UK; 3 Royal Victoria Infirmary, Newcastle upon Tyne, Tyne and Wear, UK

**Keywords:** stimulation, plasticity, rehabilitation, ARAT, upper limb, hand

## Abstract

*Background.* In monkey, reticulospinal connections to hand and forearm muscles are spontaneously strengthened following corticospinal lesions, likely contributing to recovery of function. In healthy humans, pairing auditory clicks with electrical stimulation of a muscle induces plastic changes in motor pathways (probably including the reticulospinal tract), with features reminiscent of spike-timing dependent plasticity. In this study, we tested whether pairing clicks with muscle stimulation could improve hand function in chronic stroke survivors. *Methods.* Clicks were delivered via a miniature earpiece; transcutaneous electrical stimuli at motor threshold targeted forearm extensor muscles. A wearable electronic device (WD) allowed patients to receive stimulation at home while performing normal daily activities. A total of 95 patients >6 months poststroke were randomized to 3 groups: WD with shock paired 12 ms before click; WD with clicks and shocks delivered independently; standard care. Those allocated to the device used it for at least 4 h/d, every day for 4 weeks. Upper-limb function was assessed at baseline and weeks 2, 4, and 8 using the Action Research Arm Test (ARAT), which has 4 subdomains (Grasp, Grip, Pinch, and Gross). *Results.* Severity across the 3 groups was comparable at baseline. Only the paired stimulation group showed significant improvement in total ARAT (median baseline: 7.5; week 8: 11.5; *P* = .019) and the Grasp subscore (median baseline: 1; week 8: 4; *P* = .004). *Conclusion.* A wearable device delivering paired clicks and shocks over 4 weeks can produce a small but significant improvement in upper-limb function in stroke survivors.

## Introduction

Stroke incidence has more than doubled in low- and middle-income countries in the past 3 decades.^1^ Among survivors, 30% to 66% lose the upper-limb functions^2,3^ fundamental to activities of daily living. Rehabilitating hand movements is, thus, essential to restore independence. Standard therapist-led approaches can be effective, but access to such resources in low- and middle-income countries such as India is extremely limited.^4^ Even in the United Kingdom, input from a therapist is rare beyond 6 months poststroke^5^; half of UK stroke survivors consider available rehabilitation services as suboptimal.^6^ Effective solutions that complement conventional approaches and reduce the contact time required from therapists are clearly needed if the outlook for stroke survivors, globally, is to improve.

In primates such as humans, motor control is dominated by the corticospinal tract, which is responsible for our sophisticated motor repertoire, including fine control of independent finger movements. Other pathways such as the reticulospinal tract (RST) also contribute, even to the control of the hand.^7^ The RST becomes especially important during motor recovery after corticospinal damage such as following stroke, when reticulospinal connections strengthen,^8,9^ partly subserving recovery,^10^ but also limiting the quality of recovered movements.^8,11^ Noninvasive methods to activate and manipulate the RST are limited, but in monkeys, we have shown that loud auditory clicks produce a robust burst of firing in reticular cells.^12^ We previously developed a prototype wearable device capable of continually delivering clicks paired with transcutaneous electrical stimulation of a muscle while a participant went about their normal daily activities.^13^ In healthy human volunteers, this device induced long-term changes in motor output; the direction of changes (facilitation vs suppression) depended on the click-shock interval, as expected if the stimuli induced spike-timing–dependent plasticity^14^ in the RST. We, therefore, hypothesized that this paired stimulation protocol could further strengthen RST connections in patients recovering from stroke, yielding improvements in upper-limb function.

## Aims

Supported by these recent observations, we developed the wearable device further to be suitable for patient use, with the aim of delivering a domiciliary aid to long-term rehabilitation. Here, we present the results of a clinical trial exploring the feasibility, safety, and efficacy of this device in stroke survivors with upper-limb impairment.

## Methods

### Participants

In this observer-blind, randomized, parallel-group clinical trial, consecutive stroke patients attending the neurology outpatient department and/or the stroke clinic of a regional neurosciences hospital in Kolkata, India, were assessed for their suitability for the study. We recruited patients with either hemorrhagic or ischemic hemiparetic stroke, with residual upper-limb weakness at 6 months or later from stroke onset. Patients were excluded if they had any form of aphasia, frank dementia, hearing or visual impairment, or stroke in the pontomedullary region; received electrical stimulation as part of their physical therapy; or had fixed flexor deformities of the wrist joint.

A total of 95 patients were recruited. All continued to receive standard treatment; they were randomized to receive 1 of 3 interventions: Paired Stimulation Group, wearable device delivering clicks and shocks paired at a fixed interval; Random Stimulation Group, wearable device delivering clicks and shocks at the same rate, but at random with respect to each other; and Standard Treatment Group, no device. The protocol was approved by the Institutional Ethics Committee (Reference Number INK/EthicsComm/46/2016; dated April 2, 2016), and written informed consent was taken. The protocol was registered with the Clinical Trial Registry of India (CTRI/2018/03/012628).

### Assessments

The outcomes were assessed at baseline (day 0, prior to randomization to group), week 2, week 4, and week 8 by a blinded assessor, separate from the study team member who dispensed the device to the patients. All assessments were performed by a single assessor (SC) throughout the study.

The primary outcome measure was the Action Research Arm Test (ARAT) for estimation of upper-limb function.^15^ This is a summated rating scale with 4 domains: Grasp, Grip, Pinch, and Gross. Scoring was based on the performance of a number of tasks from each domain. Each task was rated from 0 to 3, where higher scores denote less disability. There are 19 items in the scale, giving a maximum possible score of 57.

The tone of the forearm flexor group of muscles during passive extension of the wrist was assessed using the modified Ashworth Scale, which evaluates resistance to passive movement on a score from 0 to 4.^16^ Increased scores indicate increased tone; although this can be a result of spasticity, dystonia, muscle shortening, or joint contractions, after stroke spasticity is the major contributor.

The power and pinch grip strength were measured as the average of 3 measurements with electrodynamometers (G200 and P200, Biometrics Ltd, Newport, UK) of both the affected and less-affected upper limbs.

The active range of movement around the wrist joint was measured using an electrogoniometer (SG75, Biometrics Ltd, Newport, UK). The participants were requested maximally to extend and then flex their wrist from their neutral position, yielding measures of maximum active extension and flexion and total range of movement at the wrist.

Maximum contraction force about the wrist joint was measured using a custom device. Patients sat comfortably in a fixed chair, with the forearm and wrist midpronated, the hand clamped between 2 vertical plates, and the forearm strapped to a cushioned cast. The elbow was held at around 90° of flexion and the shoulder in approximately 30° of abduction. A strain gauge measured torque in the direction of wrist flexion-extension, about an axis concentric with the wrist joint. Participants were asked to make maximal contractions in flexion and extension 3 times, and the maximum values were analyzed.

Signals from power/pinch dynamometers, the goniometer, and the wrist strain gauge were digitized (power 1401 interface running Spike2 software, Cambridge Electronic Design, Cambridge, UK) and stored to hard disc for subsequent off-line analysis. The subjective feeling of satisfaction of the patient after using the device was estimated by a 5-point Likert scale (*very satisfied* to *very dissatisfied*).

### Study Procedure

After taking consent and completing the baseline (week 0) assessment, participants were randomized to 1 of the 3 groups. Randomization was performed using a customized MATLAB program. When the sequentially assigned participant number was input, the program reported whether the participant was to be issued a device (Paired Stimulation Group or Random Stimulation Group) or not (Standard Treatment Group). For the Paired or Random Stimulation Groups, the program then generated a coded file, which was copied to a microSD card and inserted into the device before issue. This instructed the device on how to configure the stimulation. Randomization and issuing of the devices was performed by a member of the team who was not involved in assessments; this person also fielded any telephone queries from the patients about device function. Patients were instructed not to discuss their device with those carrying out assessments. These procedures meant that all team members and the patients were blinded to whether patients issued with devices were in Paired or Random Stimulation Groups. Those carrying out assessments were completely blind as to group allocation. Randomization was performed block wise: for every 3 sequentially recruited patients, 1 was assigned to each group.

**Figure 1.**
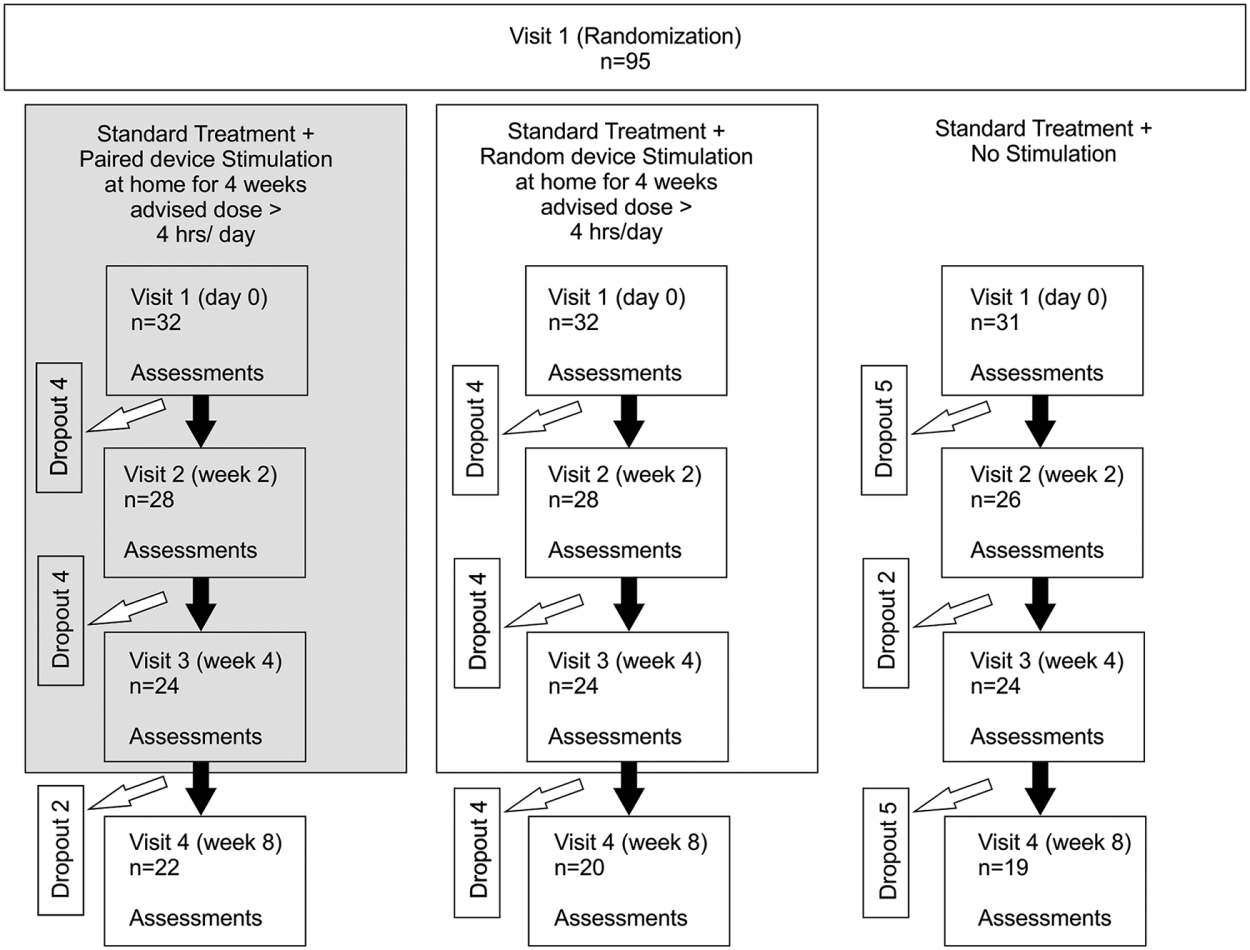
Consort diagram indicating the progress of patients from recruitment to completion of the study. Assessments used were the Action Research Arm Test, Modified Ashworth Scale, range of movement around the wrist joint, maximum wrist flexion/extension force, power and pinch grip strength (n, sample size).

Paired and Random Stimulation Groups were instructed to use the device over 4 weeks for at least 4 h/d at home from the first day of assessment (see Figure 1). Patients were told not to use the device when taking a shower or while sleeping but were otherwise free to use it for more than 4 h/d if they wished. The patients were on stable doses of medication and physical therapy from 15 days prior to the baseline visit until the end of the study visits. They were instructed to report immediately any medical occurrence during the study period. All adverse events were recorded and treated appropriately and further assessed for causality.

### Investigational Device

**Figure 2.**
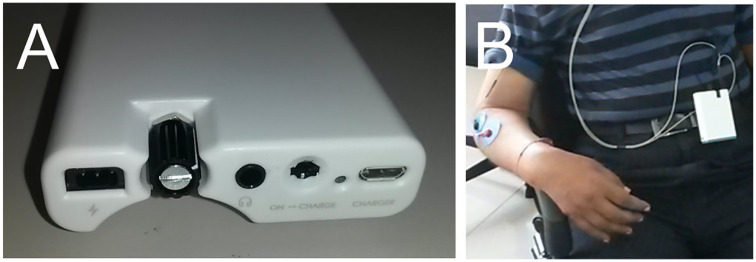
The experimental device: A. Photograph of the device, showing (from left to right) the connector for stimulating electrodes, knob for adjusting stimulus intensity, audio output to earpiece, switch to select between “on” and “charge,” LED to indicate when battery is fully charged, and micro-USB connector for charger. B. Device in use by a stroke patient in Kolkata as part of the trial to improve hand function.

The device comprised a plastic box (90 × 60 × 20 mm; see Figure 2A) containing an electrical stimulator (constant current, 220 V compliance) and audio amplifier. An inbuilt microprocessor read the SD card to determine whether to deliver paired or random stimulation and also wrote files to the SD card logging the number of stimuli given in each session. The device was powered by an internal battery that could be recharged via a standard microUSB port. Cables led from the device to a miniature earphone that delivered loud clicks (0.1-ms pulse duration; intensity 110 dB SPL as in our previous study, which should be consulted for safety calculations^17^) to the ear contralateral to the affected side and to a pair of adhesive surface electrodes (Kendall H34SG) placed over the forearm extensor muscles for transcutaneous electrical stimulation (single 0.15-ms pulse, proximal electrode negative^17^). The patients and/or their immediate family members were trained regarding the placement of electrodes until they were confident in achieving reproducible positioning. To ensure that patients/carers were continuing to place electrodes accurately, this training process was repeated at each visit. A knob on the device allowed adjustment of stimulus intensity; patients were told to increase the intensity until there was a just-visible extension of the wrist and/or fingers. Stimuli were given with an interstimulus interval randomly chosen (uniform distribution) from 1250 to 1750 ms. For the Paired Stimulation Group, each shock was given 12 ms before the click. For the Random Stimulation Group, the click and shock occurred independently at random, with the same interval distribution as in the Paired Stimulation Group.

### Statistical Analysis

During study design, limited data were available to perform a power calculation to determine optimal sample size. Therefore, 95 consecutive patients were recruited over 1 year and 7 months. Measurements from digitized force and wrist angle signals were made using custom MATLAB programs. Numerical data were presented as means and SDs (for parametric data) or medians and interquartile ranges (for nonparametric data). Categorical data were presented as percentages. Normality was assessed using a 1-sample K-S test and visual inspection of the distribution histogram and Q-Q plot, and parametric or nonparametric tests were selected accordingly. For comparing 2 groups, we used unpaired *t*-tests for parametric data and Mann-Whitney *U* tests for nonparametric data. The nonparametric data of more than 2 time points for the same patients were compared using the Friedman ANOVA with post hoc Dunn tests. The difference between 2 or more rates/proportions was compared using the Fisher exact test. The correlation between 2 numerical variables was assessed with the Spearman correlation coefficient (ρ). All participants who completed at least 1 follow-up visit were included for analysis. Intention-to-treat analysis was used. However, missing data resulting from dropout of participants were not replaced by last observed outcome. The statistical analysis was performed using SPSS version 20 (IBM Corporation).

## Results

The demographic and disease characteristics of the 95 recruited patients are presented in Table 1. Factors likely to influence motor recovery after stroke were comparable among the 3 randomized groups. There was no significant difference in the total ARAT score across the 3 groups at baseline (median in Paired Stimulation, Random Stimulation, and Standard Treatment Groups 7.5, 5, and 7 respectively; *P* = .194). There was no significant difference between the number of stimuli given in the Paired Stimulation or Random Stimulation Groups (292 000 ± 149 000 vs 243 000 ± 146 000 stimuli, respectively, mean ± SD; *P* = .251).

**Table 1.** Summary and Intergroup Comparison of Demographic and Baseline Characteristics.^a^

	Paired	Random	Standard	*P* Value
Age in years (SD)	51 (12.1)	53 (9.9)	53 (10.6)	.746
Male (%)	24 (35.8)	25 (37.3)	18 (26.9)	.191
Duration in months from onset of stroke (SD)	55 (142)	43 (94)	30 (29)	.630
Infarct (%)	19 (59.4)	20 (62.5)	19 (61.3)	1.000
Stable dose of baclofen in mg (SD)	20.4 (20.8)	15.6 (12.1)	15.6 (13.6)	.474
Physiotherapy hours per week (SD)	4.2 (2.6)	3.5 (2.2)	6.1 (3.7)	.203
Mean ARAT at baseline (SD)	18.3 (19.4)	10.8 (12.3)	17.3 (20.0)	.194
Modified Ashworth score, mean (SD) at baseline	1.5 (0.8)	1.9 (1.0)	2.1 (1.2)	.179
Mean power grip at baseline, affected as percentage of unaffected (SD)	28.9 (22.98)	22.7 (13.16)	30.28 (16.48)	.216
Range of movement around wrist in degrees (SD)	48.4 (40.21)	30.23 (37.06)	42.82 (43.59)	.315
MCA (%)	13 (40.6)	16 (50.0)	16 (51.6)	.884
ACA (%)	1 (3.1)	1 (3.1)	1 (3.2)	
PCA (%)	3 (9.4)	1 (3.1)	1 (3.2)	

Abbreviations: ACA, anterior cerebral artery; ARAT, Action Research Arm Test; MCA, middle cerebral artery; PCA, posterior cerebral artery.

a The difference of numerical variables among the 3 independent groups was estimated using 1-way ANOVA for parametric data, Kruskal-Wallis H test for nonparametric data, and categorical variables using the Fisher exact test. *P* value <.05 was considered statistically significant.

The Paired Stimulation Group showed a significant effect of visit number on total ARAT score (*P* = .019); post hoc pairwise testing showed an improvement from visit 1 to both visits 3 and 4 (median ARAT 7.5, 12.5, and 11.5, respectively; *P* = .012 and *P* = .023; Kendall *W* for visit number used as an estimate of effect size of 0.15). The Random Stimulation and Standard Treatment Groups showed no effect of visit number on the total ARAT scores (see Table 2).

**Table 2.** Change in ARAT Score and ARAT Subscores Over a Period of 8 Weeks.^a^

	V1-ARAT	V2-ARAT	V3-ARAT	V4-ARAT	*P* Value
Paired	7.5 (3.25-30.5)	11 (5-38)	12.5 (4.5-33.5)^b^	11.5 (5-33.5)^b^	.019^b^
Random	5 (3-15)	6.5 (4-20.75)	8.5 (4-15)	7.5 (0.25-18.75)	.071
Standard	7 (1-32)	10 (1.5-24.75)	12 (3-31)	9 (0-21)	.794
	V1-Grasp	V2-Grasp	V3-Grasp	V4-Grasp	*P* Value
Paired	1 (0-12)	3.5 (0-14.25)	5 (0-13.25)	4 (0-14)^b^	.004^b^
Random	1 (0-8)	2 (0-9.25)	2.5 (0-6.75)	3 (0-10)	.079
Standard	1 (0-11)	10 (1.5-24.75)	4 (0-11.75)	4 (0-8.5)	.479
	V1-Grip	V2-Grip	V3-Grip	V4-Grip	*P* Value
Paired	2 (0-7)	3 (1-7)	2.5 (0.5-6.75)	3 (0.75-14)	.102
Random	0.5 (0-3.75)	2 (0-4.75)	2 (0-4)	2 (0-4)	.247
Standard	2 (0-6)	2.5 (0-7)	3 (0-7.75)	3.5 (0-5.5)	.923
	V1-Pinch	V2-Pinch	V3-Pinch	V4-Pinch	*P* Value
Paired	0 (0-8.25)	0 (0-11.5)	0 (0-9)	0 (0-8.25)	.055
Random	0 (0-0)	0 (0-1)	0 (0-1)	0 (0-1)	.050
Standard	0 (0-8)	0 (0-2.75)	0 (0-8)	0 (0-1.25)	.491
	V1-Gross	V2-Gross	V3-Gross	V4-Gross	*P* Value
Paired	3.5 (3-6)	4 (3-7.75)	4 (3-6.75)	3 (3-4.75)	.121
Random	3 (3-5.5)	3.5 (3-6)	4 (3-5.75)	3 (0-4)	.215
Standard	4 (0-6)	3.5 (0-6)	3.5 (3-6)	3.5 (1.5-6)	.324

Abbreviation: ARAT, Action Research Arm Test.

a The difference of numerical variables expressed as median (interquartile range) in multiple time points for the same patients was estimated using the Friedman ANOVA. Pairwise comparison was by the post hoc Dunn test with the Friedman ANOVA.

b *P* value <.05 was considered statistically significant.

The above analysis reports changes at a population level; it was also of interest to look at how many individual patients showed an improvement. We defined *responders* as patients with at least a 6-point increase in the total ARAT score (~10% of maximum). Seven patients (29%) from the Paired Stimulation Group compared with 1 (4%) of each of the other 2 groups were responders at visit 3; these proportions were significantly different (*P* = .015). The response persisted at visit 4 in 5/7 responders from the Paired Stimulation Group.

The Grasp subscore improved significantly only in the Paired Stimulation Group. The Grip, Pinch, and Gross subscores did not show any significant change in any of the study groups (Table 2). Other outcome parameters (modified Ashworth score, isometric grip and wrist strength, angular movement around the wrist) did not show significant changes in any group (see Supplementary Information).

Age, sex, duration since onset of stroke, type of stroke (hemorrhagic/ischemic infarction), affected side (left/right), and median baseline ARAT total score and subscores were not significantly different between responders and nonresponders, as classified using visit 3 scores. The total number of paired stimuli received over the 4 weeks of device use was significantly correlated with the change in ARAT score at visit 3 (Spearman ρ = 0.53; *P* = .013) but not at visit 4 (ρ = 0.285; *P* = .223). There was no correlation between the number of stimuli received in the Random Stimulation group and ARAT change (Spearman ρ = −0.052, *P* = .814, and ρ = 0.053, *P* = .835, for visits 3 and 4, respectively).

Trial participants reported that stimulation did not interfere with or interrupt their normal activities of daily living, which typically involved light household work or leisure activities. Only 1 patient experienced a device-related adverse event. This individual developed a contact dermatitis where the adhesive electrodes had been placed. This improved with topical steroid application and did not have an impact on the experimental intervention because electrodes could easily be relocated to avoid the skin lesion. All patients successfully used the prototype device, although the study identified that the micro-USB charge point was weak and prone to breakage (4 devices over the entire study duration). Two patients disliked the repeated click sound and withdrew from the study at visit 2. Of 64 (73%) patients who received a device intervention, 47 were either very satisfied or satisfied with the intervention. Of 22 patients who withdrew from the study by visit 3 (device users), the majority (14) did so because of the burden of long-distance travel to our hospital from their place of residence.

## Discussion

In this clinical trial, we observed that the Paired Stimulation Group demonstrated a small but statistically significant improvement of upper-limb function over the 4 weeks of device use, which was retained for at least 4 weeks after device stimulation ceased. Within this group, the extent of functional gain was correlated with stimulus number: those patients who chose to use the stimulation device for longer each day had better functional improvement. In contrast, patients allocated to the control groups (Random Stimulation or Standard Treatment) did not show a significant improvement, suggesting that the benefit results specifically from paired stimulation.

Various neuromuscular stimulation modalities have been previously used for upper- and lower-limb motor recovery after stroke.^18-20^ Either these devices are used to enhance a weak voluntary movement (functional electrical stimulation)^21^ or stimulate muscles in the absence of any simultaneous effort from the patient.^22^ These devices have been found to be useful in the majority of clinical trials, although the improvement is usually only apparent during the spontaneous recovery phase.^23,24^

Loud sounds are known to be capable of activating not just the cochlea, but also the vestibular system; muscle responses to loud clicks are used routinely for the assessment of the functional integrity of vestibular pathways (vestibular evoked myogenic potentials, see Rosengren et al^25^). Previous work has shown that vestibular rehabilitation,^26-29^ rhythmic auditory stimulation,^30^ and music therapy^31^ can all improve gait in stroke survivors. Extrapyramidal pathways such as the vestibulospinal tract and RST receive strong vestibular and auditory inputs and are intimately associated with the control of posture and locomotion; a contribution from the brainstem to recovery of gait might, therefore, be expected. However, we have shown that the RST also contributes to recovery of upper-limb function after corticospinal tract damage.^7,10,32^ To date, no studies have considered whether vestibular or auditory stimuli might improve rehabilitation of hand movements. Loud clicks can powerfully activate reticulospinal cells,^12^ and pairing clicks with peripheral stimuli can induce long-lasting changes in motor output consistent with spike-timing–dependent plasticity.^17^ These 2 observations led us to the present trial, which represents a novel and unique approach to stimulation-based therapy.

Our randomized, observer-blind clinical trial demonstrated a significant improvement in total ARAT score and the Grasp subscore following paired click and shock stimulation. This represents high-quality evidence of a benefit at a population level, but on average, the changes were small (5-point median change in ARAT). In a chronic hemiparetic population, the patient-perceived minimal clinically important improvement has been estimated as 5.7 points^33^ (10% of the maximum possible ARAT score). However, here, there was considerable interindividual variation in the extent of improvement. Better outcomes were associated with higher baseline grip strength in the affected hand and also with using the stimulation device for longer each day, thereby delivering more paired stimuli. Thus, when deciding whether to use our device for treatment, patients and their caregivers should be informed that only a subset of individuals with specific baseline characteristics demonstrated a significant outcome following 4 weeks of treatment. Future trials must address whether longer durations of device intervention beyond the minimum 4 h/d for 4 weeks tested here could extend functional benefits to a wider group of patients. It would also be of interest to combine this protocol with other stimulation paradigms that may access different pathways^34,35^ because this could allow synergistic gains in function.

Recent work in spinal cord injury survivors suggests that high spasticity is associated with limited residual corticospinal connections and enhanced reticulospinal output below the lesion^36,37^; this accords with clinical experience associating spasticity with the RST.^38^ Against this background, it might be thought that our intervention, which aimed to strengthen reticulospinal outputs, could have risked increasing spasticity. Reassuringly, our results yielded no evidence of increases in spasticity as measured by the Modified Ashworth Score.

There is evidence that spontaneous recovery of hand function after stroke relies on 2 separable systems: one provides strength and a limited degree of digit fractionation, whereas the other adds further ability to generate independent finger movements.^39^ The system mainly responsible for strength recovery may be associated with the RST. This would agree with a recent study in our laboratory, where we have revealed a reticulospinal contribution to neural adaptations following strength training in healthy monkeys.^40^ Despite this, in the present work, we found no change in grip or wrist strength in the Paired Stimulation Group.

It is possible that the paired click and shock stimulation exerted its effects on systems other than the RST; this could explain why we observed no change in spasticity or strength. However, it is likely that the RST has multiple subdivisions, based on the reticular nucleus of origin^41^ and the laterality of both projections to the cord, and control from the cortex.^42^ This richness probably explains why enhanced reticulospinal outputs have been associated by different authors with both recovery^10,32,43^ and poor outcomes.^11,44^ It is possible that the paired stimulation used here accessed only a subset of reticulospinal outputs, yielding an overall positive benefit to hand function. It is also possible that changes were too small to generate overt increases in strength but still sufficient to yield improved control and enhanced ARAT scores.

Examination of the subcomponents of the ARAT test showed a significant improvement in Grasp, but not in Grip, Pinch, or Gross subscores. An improvement in hand movements requiring less well-fractionated muscle activation (Grasp) rather than fine independent finger movements (Grip, Pinch) would be compatible with a contribution from the reticulospinal rather than corticospinal tracts.^45,46^ The lack of effect on the Gross subscore may reflect the fact that we targeted a forearm extensor muscle for stimulation, rather than more proximal muscles. Spontaneous recovery of hand function after stroke is typically imbalanced. Whereas wrist and digit flexors often regain strength, the extensors remain weak, contributing substantially to disability.^47^ This mirrors the pattern of spontaneous changes in reticulospinal connections after corticospinal lesions in monkeys: outputs are strengthened to flexors but not to extensors.^10^ We recently found that some forms of paired associative stimulation showed a similar bias in their ability to induce plasticity in the corticospinal tract. No matter whether flexors or extensor muscles were stimulated, outputs were enhanced to flexors but not extensors.^35^ Although we targeted the forearm extensor muscles in the present trial, we found no change in isometric wrist extension strength in any group. Likewise, there was no change in active range of movement around the wrist, which is most affected in stroke survivors by extensor weakness. This may indicate that, just like the corticospinal tract, the RST has only a limited ability for stimulus-induced plasticity in output to extensors. The fact that there was, nevertheless, an average improvement in hand function hints at a complex reorganization of control pathways, rather than a simple enhancement of 1 component.

One limitation of our study design was that we could not standardize the physical therapy program across patients. Many patients were receiving little or no physical therapy. However, there were no intergroup differences in the frequency or duration of physical therapy, suggesting that this could not have affected our results. We would expect even greater functional gains if the stimulation device was used concomitantly with a customized therapy regime, ideally at high dosage.^48^ In this trial, we targeted chronic stroke patients to avoid the difficulty of trying to detect benefit against a moving baseline. The lack of significant changes in the control groups confirmed that spontaneous recovery had indeed largely ceased. Stroke seems to generate a short-lived window of enhanced plasticity in the acute and subacute phases.^49^ Using our paired stimulation device during this window might lead to even greater gains than we have seen in chronic patients, where plasticity has likely returned to baseline levels.

## Supplemental Material

NNR_34_7 – Supplemental material for A Novel Wearable Device for Motor Recovery of Hand Function in Chronic Stroke Survivors Supplemental material, NNR_34_7 for A Novel Wearable Device for Motor Recovery of Hand Function in Chronic Stroke Survivors by Supriyo Choudhury, Ravi Singh, A. Shobhana, Dwaipayan Sen, Sidharth Shankar Anand, Shantanu Shubham, Suparna Gangopadhyay, Mark R. Baker, Hrishikesh Kumar and Stuart N. Baker in Neurorehabilitation and Neural Repair

Supppl_doc – Supplemental material for A Novel Wearable Device for Motor Recovery of Hand Function in Chronic Stroke Survivors Supplemental material, Supppl_doc for A Novel Wearable Device for Motor Recovery of Hand Function in Chronic Stroke Survivors by Supriyo Choudhury, Ravi Singh, A. Shobhana, Dwaipayan Sen, Sidharth Shankar Anand, Shantanu Shubham, Suparna Gangopadhyay, Mark R. Baker, Hrishikesh Kumar and Stuart N. Baker in Neurorehabilitation and Neural Repair
