# Supplementary figures and images for: A Novel Wearable Device for Motor Recovery of Hand Function in Chronic Stroke Survivors
Source: Neurorehabil Neural Repair. 2020 May 26;34(7):600–8. doi: 10.1177/1545968320926162 (PMC8207486; doi:10.1177/1545968320926162)

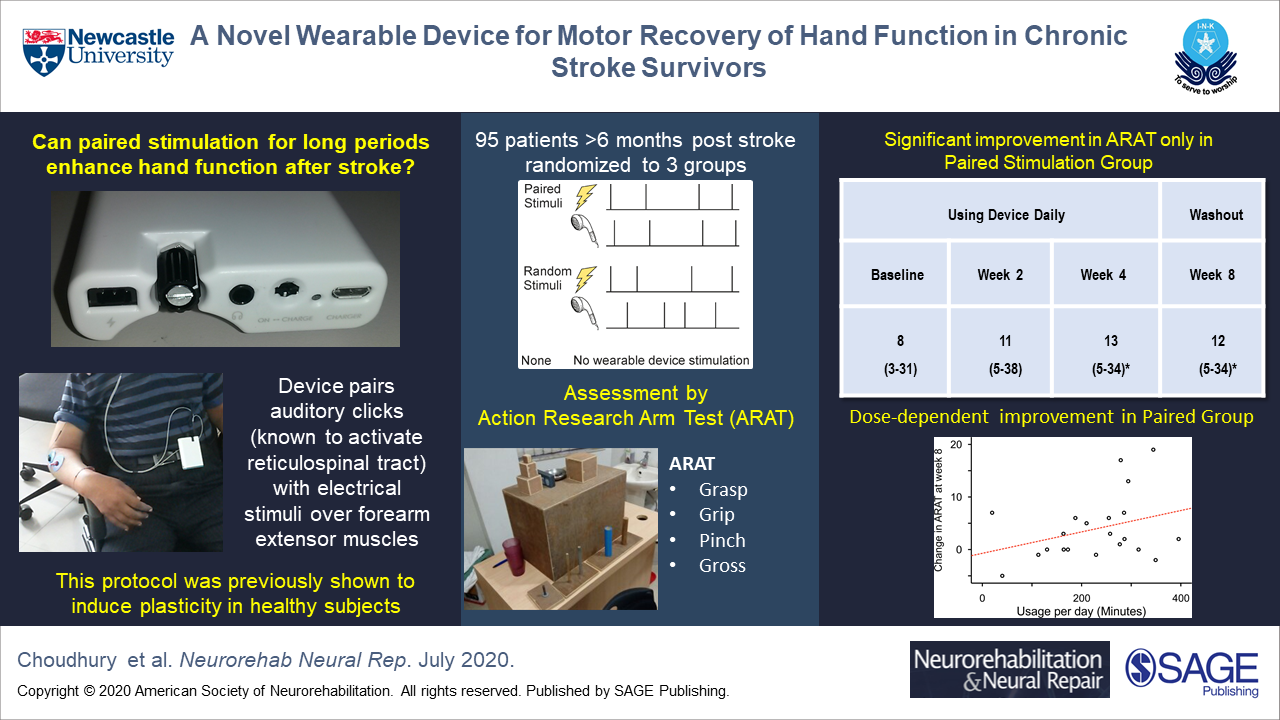

Supplement: NNR_34_7 – Supplemental material for A Novel Wearable Device for Motor Recovery of Hand Function in Chronic Stroke Survivors [file NNR_34_7.png]
