# Supplementary material for: A Novel Wearable Device for Motor Recovery of Hand Function in Chronic Stroke Survivors
Source: Neurorehabil Neural Repair. 2020 May 26;34(7):600–8. doi: 10.1177/1545968320926162 (PMC8207486; doi:10.1177/1545968320926162)
Supplement: Supppl_doc – Supplemental material for A Novel Wearable Device for Motor Recovery of Hand Function in Chronic Stroke Survivors [file Supppl_doc.docx]

**Supplementary table 1: Summary and inter-group comparison of ARAT scores in consecutive time points.** The difference of numerical variables expressed as mean (standard deviation) and median (interquartile range) in multiple groups were estimated using Kruskal-Wallis ANOVA. P value < 0.05 was considered statistically significant.

| **Baseline ARAT** | | | | |
| --- | --- | --- | --- | --- |
|  | Paired | Random | Standard | P value |
| **Mean** | 18.09 | 10.75 | 17.26 | 0.198 |
| **Median** | 7.500 | 5.000 | 7.000 |  |
| **Std. Deviation** | 19.33 | 12.50 | 20.27 |  |
| **25th percentile** | 3.250 | 3.000 | 1.000 |  |
| **75th percentile** | 30.50 | 15.00 | 32.00 |  |
| **Week 2 ARAT** | | | | |
|  | Paired | Random | Standard | P value |
| **Mean** | 20.79 | 13.29 | 16.50 | 0.299 |
| **Median** | 11.00 | 6.500 | 10.00 |  |
| **Std. Deviation** | 20.39 | 13.76 | 19.19 |  |
| **25th percentile** | 5.000 | 4.000 | 1.500 |  |
| **75th percentile** | 38.00 | 20.75 | 24.75 |  |
| **Week 4 ARAT** | | | | |
|  | Paired | Random | Standard | P value |
| **Mean** | 20.46 | 13.46 | 18.33 | 0.396 |
| **Median** | 12.50 | 8.500 | 12.00 |  |
| **Std. Deviation** | 19.65 | 15.12 | 19.34 |  |
| **25th percentile** | 4.500 | 4.000 | 3.000 |  |
| **75th percentile** | 33.50 | 15.00 | 31.00 |  |
| **Week 8 ARAT** | | | | |
|  | Paired | Random | Standard | P value |
| **Mean** | 19.77 | 12.00 | 14.42 | 0.310 |
| **Median** | 11.50 | 7.500 | 9.000 |  |
| **Std. Deviation** | 19.00 | 14.17 | 16.38 |  |
| **25th percentile** | 5.000 | 0.2500 | 0.000 |  |
| **75th percentile** | 33.50 | 18.75 | 21.00 |  |

**Supplementary table 2: Summary and inter-group comparison of grasp sub-scores in consecutive time points.** The difference of numerical variables expressed as mean (standard deviation) and median (interquartile range) in multiple groups were estimated using Kruskal-Wallis ANOVA. P value < 0.05 was considered statistically significant.

| **Baseline Grasp** | | | | |
| --- | --- | --- | --- | --- |
|  | Paired | Random | Standard | P value |
| **Mean** | 5.844 | 3.531 | 5.452 | 0.709 |
| **Median** | 1.000 | 1.000 | 1.000 |  |
| **Std. Deviation** | 7.030 | 4.879 | 6.707 |  |
| **25th percentile** | 0.000 | 0.000 | 0.000 |  |
| **75th percentile** | 12.00 | 8.000 | 11.00 |  |
| **Week 2 Grasp** | | | | |
|  | Paired | Random | Standard | P value |
| **Mean** | 6.679 | 4.571 | 5.538 | 0.795 |
| **Median** | 3.500 | 2.000 | 2.500 |  |
| **Std. Deviation** | 7.409 | 5.718 | 6.730 |  |
| **25th percentile** | 0.000 | 0.000 | 0.000 |  |
| **75th percentile** | 14.25 | 9.250 | 11.00 |  |
| **Week 4 Grasp** | | | | |
|  | Paired | Random | Standard | P value |
| **Mean** | 6.875 | 4.542 | 6.208 | 0.643 |
| **Median** | 5.000 | 2.500 | 4.000 |  |
| **Std. Deviation** | 7.067 | 5.524 | 6.541 |  |
| **25th percentile** | 0.000 | 0.000 | 0.000 |  |
| **75th percentile** | 13.25 | 6.750 | 11.75 |  |
| **Week 8 Grasp** | | | | |
|  | Paired | Random | Standard | P value |
| **Mean** | 7.182 | 5.053 | 5.278 | 0.652 |
| **Median** | 4.000 | 3.000 | 4.000 |  |
| **Std. Deviation** | 7.228 | 5.602 | 5.809 |  |
| **25th percentile** | 0.000 | 0.000 | 0.000 |  |
| **75th percentile** | 14.00 | 10.00 | 8.500 |  |

**Supplementary table 3: Summary and inter-group comparison of grip sub-scores in consecutive time points.** The difference of numerical variables expressed as mean (standard deviation) and median (interquartile range) in multiple groups were estimated using Kruskal-Wallis ANOVA. P value < 0.05 was considered statistically significant.

| **Baseline Grip** | | | | |
| --- | --- | --- | --- | --- |
|  | Paired | Random | Standard | P value |
| **Mean** | 4.063 | 2.219 | 3.742 | 0.222 |
| **Median** | 2.000 | 0.5000 | 2.000 |  |
| **Std. Deviation** | 4.265 | 2.992 | 4.531 |  |
| **25th percentile** | 0.000 | 0.000 | 0.000 |  |
| **75th percentile** | 7.000 | 3.750 | 6.000 |  |
| **Week 2 Grip** | | | | |
|  | Paired | Random | Standard | P value |
| **Mean** | 4.393 | 2.964 | 3.731 | 0.510 |
| **Median** | 3.000 | 2.000 | 2.500 |  |
| **Std. Deviation** | 4.280 | 3.109 | 4.387 |  |
| **25th percentile** | 1.000 | 0.000 | 0.000 |  |
| **75th percentile** | 7.000 | 4.750 | 7.000 |  |
| **Week 4 Grip** | | | | |
|  | Paired | Random | Standard | P value |
| **Mean** | 4.250 | 2.708 | 4.083 | 0.484 |
| **Median** | 2.500 | 2.000 | 3.000 |  |
| **Std. Deviation** | 4.173 | 3.099 | 4.413 |  |
| **25th percentile** | 0.5000 | 0.000 | 0.000 |  |
| **75th percentile** | 6.750 | 4.000 | 7.750 |  |
| **Week 8 Grip** | | | | |
|  | Paired | Random | Standard | P value |
| **Mean** | 4.455 | 2.947 | 3.722 | 0.552 |
| **Median** | 3.000 | 2.000 | 3.500 |  |
| **Std. Deviation** | 4.228 | 3.325 | 3.997 |  |
| **25th percentile** | 0.7500 | 0.000 | 0.000 |  |
| **75th percentile** | 8.250 | 4.000 | 5.500 |  |

**Supplementary table 4: Summary and inter-group comparison of pinch sub-scores in consecutive time points.** The difference of numerical variables expressed as mean (standard deviation) and median (interquartile range) in multiple groups were estimated using Kruskal-Wallis ANOVA. P value < 0.05 was considered statistically significant.

| **Baseline pinch** | | | | |
| --- | --- | --- | --- | --- |
|  | Paired | Random | Standard | P value |
| **Mean** | 4.000 | 1.188 | 4.032 | 0.179 |
| **Median** | 0.000 | 0.000 | 0.000 |  |
| **Std. Deviation** | 6.263 | 3.780 | 7.007 |  |
| **25th percentile** | 0.000 | 0.000 | 0.000 |  |
| **75th percentile** | 8.250 | 0.000 | 8.000 |  |
| **Week 2 pinch** | | | | |
|  | Paired | Random | Standard | P value |
| **Mean** | 4.857 | 1.893 | 3.154 | 0.250 |
| **Median** | 0.000 | 0.000 | 0.000 |  |
| **Std. Deviation** | 7.096 | 4.573 | 6.123 |  |
| **25th percentile** | 0.000 | 0.000 | 0.000 |  |
| **75th percentile** | 11.50 | 1.000 | 2.750 |  |
| **Week 4 pinch** | | | | |
|  | Paired | Random | Standard | P value |
| **Mean** | 4.417 | 2.083 | 4.000 | 0.628 |
| **Median** | 0.000 | 0.000 | 0.000 |  |
| **Std. Deviation** | 6.921 | 5.141 | 6.897 |  |
| **25th percentile** | 0.000 | 0.000 | 0.000 |  |
| **75th percentile** | 9.000 | 1.000 | 8.000 |  |
| **Week 8 pinch** | | | | |
|  | Paired | Random | Standard | P value |
| **Mean** | 3.364 | 1.368 | 2.333 | 0.985 |
| **Median** | 0.000 | 0.000 | 0.000 |  |
| **Std. Deviation** | 6.268 | 4.112 | 5.258 |  |
| **25th percentile** | 0.000 | 0.000 | 0.000 |  |
| **75th percentile** | 4.750 | 1.000 | 1.250 |  |

**Supplementary table 5: Summary and inter-group comparison of gross sub-scores in consecutive time points.** The difference of numerical variables expressed as mean (standard deviation) and median (interquartile range) in multiple groups were estimated using Kruskal-Wallis ANOVA. P value < 0.05 was considered statistically significant.

| **Baseline gross** | | | | |
| --- | --- | --- | --- | --- |
|  | Paired | Random | Standard | P value |
| **Mean** | 4.250 | 3.719 | 3.935 | 0.826 |
| **Median** | 3.500 | 3.000 | 4.000 |  |
| **Std. Deviation** | 2.615 | 2.020 | 3.183 |  |
| **25th percentile** | 3.000 | 3.000 | 0.000 |  |
| **75th percentile** | 6.000 | 5.500 | 6.000 |  |
| **Week 2 gross** | | | | |
|  | Paired | Random | Standard | P value |
| **Mean** | 4.857 | 4.214 | 3.808 | 0.510 |
| **Median** | 4.000 | 3.500 | 3.500 |  |
| **Std. Deviation** | 2.534 | 2.250 | 3.124 |  |
| **25th percentile** | 3.000 | 3.000 | 0.000 |  |
| **75th percentile** | 7.750 | 6.000 | 6.000 |  |
| **Week 4 gross** | | | | |
|  | Paired | Random | Standard | P value |
| **Mean** | 4.667 | 4.125 | 4.042 | 0.786 |
| **Median** | 4.000 | 4.000 | 3.500 |  |
| **Std. Deviation** | 2.200 | 2.213 | 2.805 |  |
| **25th percentile** | 3.000 | 3.000 | 3.000 |  |
| **75th percentile** | 6.750 | 5.750 | 6.000 |  |
| **Week 8 gross** | | | | |
|  | Paired | Random | Standard | P value |
| **Mean** | 4.773 | 3.263 | 3.889 | 0.330 |
| **Median** | 3.000 | 3.000 | 3.500 |  |
| **Std. Deviation** | 2.349 | 2.557 | 2.888 |  |
| **25th percentile** | 3.000 | 0.000 | 1.500 |  |
| **75th percentile** | 7.250 | 4.000 | 6.000 |  |
